# Supplementary figures and images for: Transcriptomic Profiling of Fibropapillomatosis in Green Sea Turtles (Chelonia mydas) From South Texas
Source: Front Immunol. 2021 Feb 24;12:630988. doi: 10.3389/fimmu.2021.630988 (PMC7943941; doi:10.3389/fimmu.2021.630988)

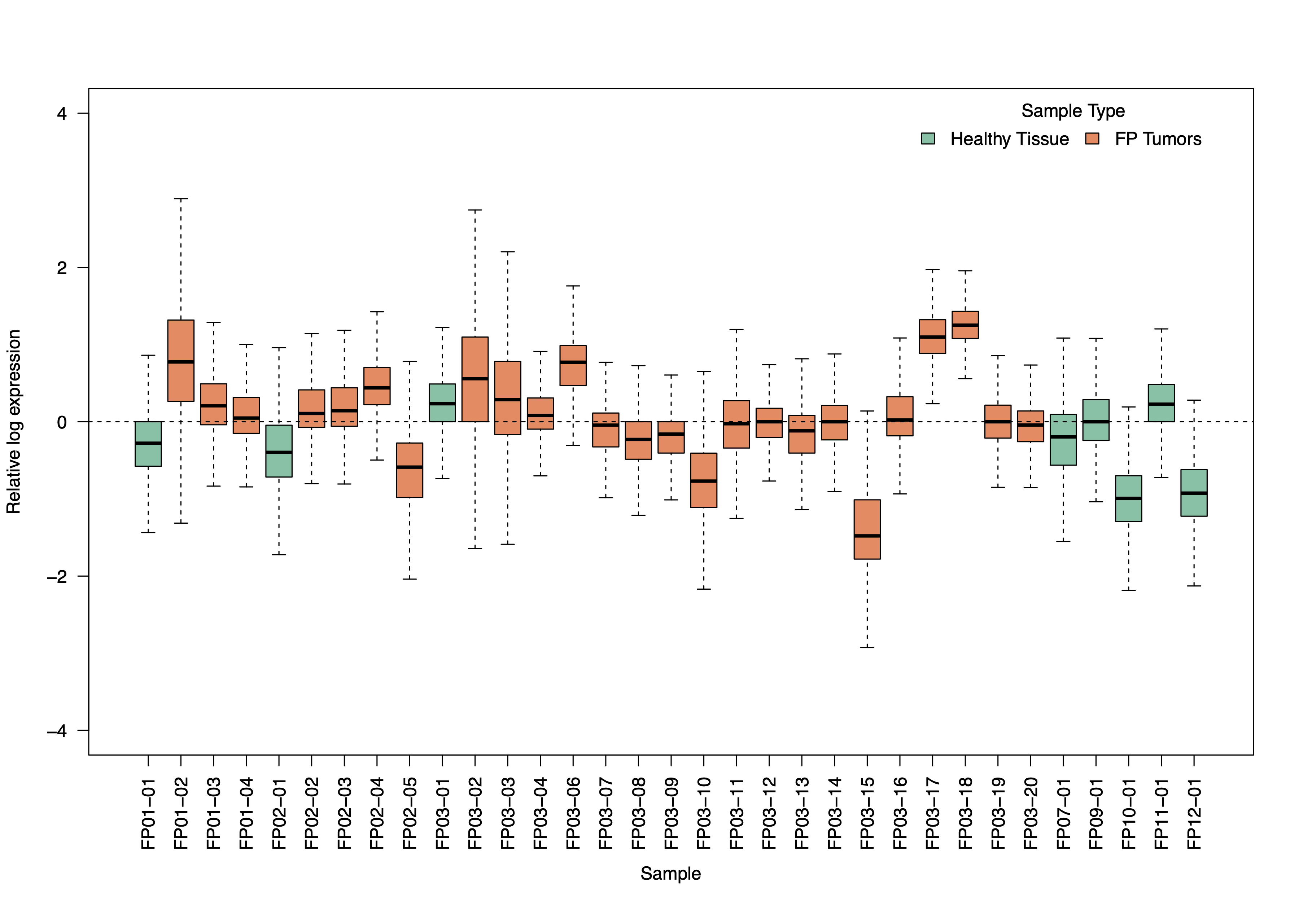

Supplement: Supplementary Figure 1 — Relative log expression plot of the gene expression data per sample prior to adjustment for two factors of unwanted variation calculated using RUVSeq. [file Image_1.jpg]

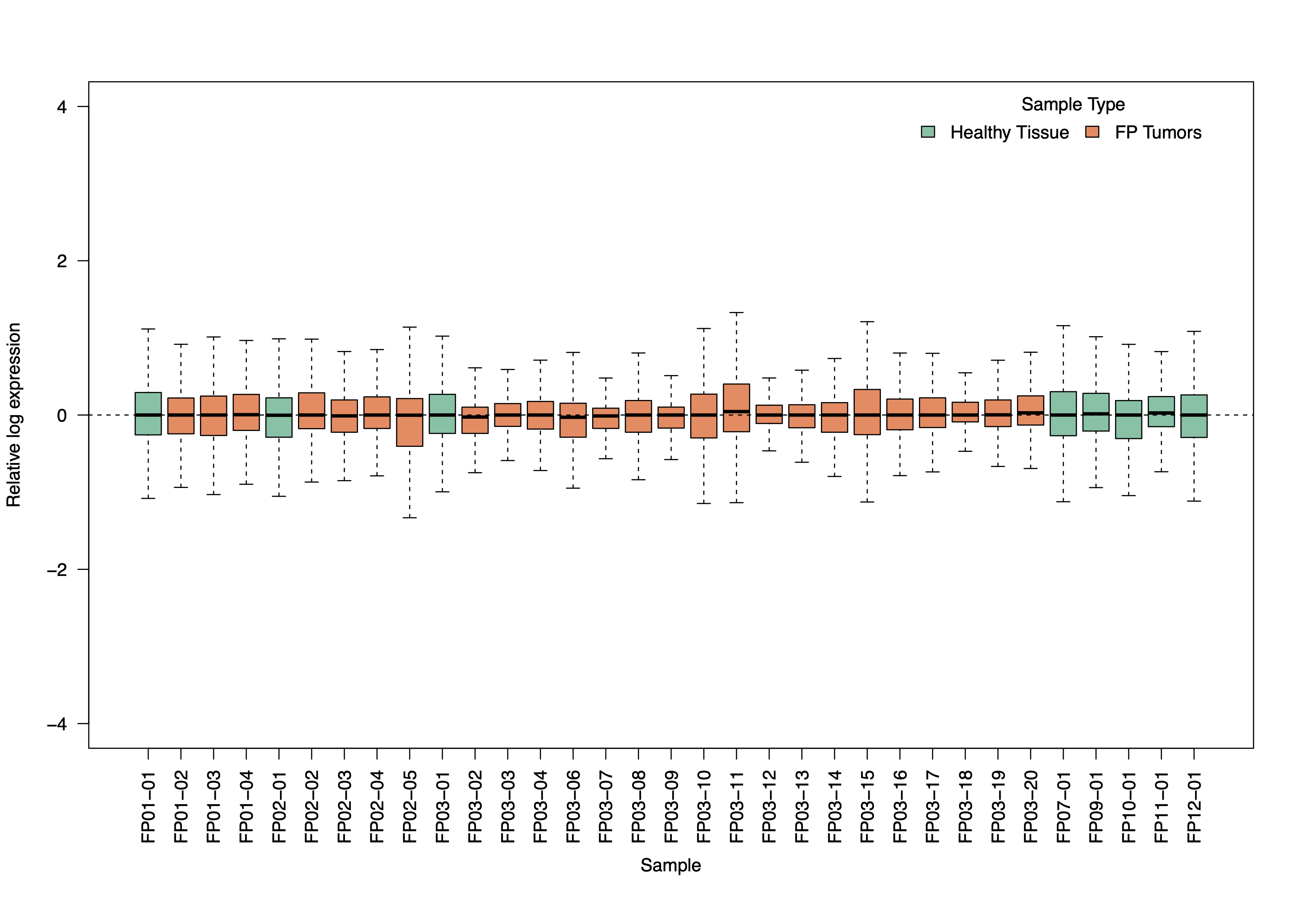

Supplement: Supplementary Figure 2 — Relative log expression plot of the gene expression data per sample after adjustment for two factors of unwanted variation calculated using RUVSeq. [file Image_2.jpg]
